# Perceived Moral Norms in an Extended Theory of Planned Behavior in Predicting University Students’ Bystander Intentions toward Relational Bullying

**DOI:** 10.3390/ejihpe13070089

**Published:** 2023-07-02

**Authors:** Mareike Brehmer

**Affiliations:** Department of Education, University of Agder, 4630 Kristiansand, Norway; mareike.brehmer@uia.no; Tel.: +47-46744808

**Keywords:** bystander, bullying, social exclusion, relational aggression, theory of planned behavior, moral norms

## Abstract

Relational forms of bullying, such as social exclusion, are prevalent among students in higher education (HE) and pose challenges to the creation of a safe and inclusive learning environment for young adults. Based on research showing how bystanders in bullying incidents can counteract bullying effectively, the present study investigated the extent to which bystanders’ prosocial behavioral intentions may be predicted using an extended theory of planned behavior (TPB). Students’ behavioral intentions toward the physical–social exclusion of peers in a hypothetical group work setting were investigated in a sample of 419 university students in the United Kingdom. Hierarchical regression analysis showed that moral norms significantly predicted intention over and above cognitive attitude, descriptive norms, and perceived behavioral control. In contrast, emotional attitudes and injunctive norms were not significant predictors of intention. Moreover, significant weak mediation effects could be shown for moral norms as a mediator of the relationships between standard TPB predictors and intention. The present study contributes knowledge to the growing research body on applications of the TPB and on bystander intentions in bullying in HE. Implications for a development in preventive measures to foster university students’ prosocial intentions toward bullying are discussed.

## 1. Introduction

Bullying generally refers to negative actions performed by one or more individuals against a weaker peer. Definitions highlight power imbalance, repeated aggression, and the victim’s inability to defend themselves [1,2,3]. While research on bullying in higher education (HE) is increasing, it represents a relatively neglected area in terms of developing nuanced knowledge [4,5]. In HE, bullying may occur in the form of gossip, mocking, or social exclusion [4,6]. When individuals are socially excluded by a group, they are likely to experience a lack of belongingness [7,8], which can have detrimental effects on their mental health [1,9,10]. Social exclusion falls under the category of relational bullying and has currently been explored in a few studies on the prevalence of bullying in HE [2,7,11]. For instance, a Norwegian study reported that approximately 10% of students experience situations of “being overlooked, purposefully ignored” or “excluded from groups or activities” [7]. While the prevalence of various forms of bullying may vary across different cultural contexts, available data consistently underscore the importance of researching bullying in HE [2,11].

A prominent aspect of bullying in HE is related to the role of bystanders’ moral dilemmas in bullying situations [12,13,14]. Bystanders should determine whether the observed behavior is bullying (and distinguish it, e.g., from banter) and may require additional information about the situation and available courses of action [6,15,16]. In addition, witnesses of bullying understand that it is wrong and feel that they assist the target individual; however, they are also concerned about their popularity and social status [17]. Hence, the need to belong and feel safe may override feelings of guilt and shame, resulting in their reluctance to involve themselves [18,19,20,21,22]. Thus, gaining an understanding of bystanders’ cognitive and emotional intentions within their moral dilemma provides knowledge regarding the underlying mechanisms influencing them to help, or not to help, victims of bullying.

### 1.1. Theory of Planned Behavior

A widely recognized theoretical framework used to explain the connection between social cognition and human behavior is Icek Ajzen’s [23] theory of planned behavior (TPB) [24]. The TPB posits that the most important predictor of behavioral performance is an individual’s intention to engage in the behavior. This intention is determined by (i) attitudes toward performing a given action, (ii) subjective normative beliefs, and (iii) perceived behavioral control. In the context of bystanders, this implies that they evaluate the situation based on their observations and determine whether their involvement can lead to a positive or negative outcome. Furthermore, bystanders relate this outcome to their peer group’s norms and assume mutual expectations on their involvement in the situation. Finally, their actions are influenced by how much control they perceive they have over the situation.

Attitudes, within the TPB, are divided into two subconstructs: emotional attitude and cognitive attitude [23]. Subjective norms refer to a measure of what would commonly be described as social or peer pressure [23]. The TPB further distinguishes between two types of underlying normative beliefs: injunctive norms, which describe the subjective probability of approval or disapproval by a significant individual or group, and descriptive norms, which refer to whether those significant others actually engage in the behavior themselves [25].

### 1.2. Extension to the Theory of Planned Behavior 

In TPB research, it has become common practice to engage in “theory broadening” to increase the number of predictors in the original model [26]. General research has demonstrated the predictive validity of moral norms on behavioral intentions [27,28]. Manstead [29] defined moral norms within the TPB as an individual’s conviction that certain ways of acting are “inherently right or wrong… regardless of their personal or social consequences” (p. 12). Considering the moral implications of bullying, incorporating perceived moral norms in this context is recommended [30]. This is because most people are aware that causing harm to others is wrong; however, despite this knowledge, some individuals either go against these norms or allow them to be violated when they witness immoral behaviors.

### 1.3. Present Study 

The general utility of the TPB in predicting intentions is evident [27,31,32], and over time, the TPB has been applied to the role of a bystander in bullying situations, including cyberbullying in HE [13,14] and school bullying [33,34,35,36]. However, to the best knowledge, one unexplored context is university students’ intentions toward social exclusion among their peers in HE. Thus, the present study has two objectives. The first objective was to examine the extent to which the TPB provides an adequate theoretical framework for predicting university students’ bystander intentions toward relational bullying. The second objective was to determine the extent to which the inclusion of perceived moral norms in an extended TPB model contributes to these predictions. Building on previous research, the following hypotheses were tested:

**H_1a–e_.**  *The traditional TPB components, including (H_1a_) emotional attitudes, (H_1b_) cognitive attitudes, (H_1c_) injunctive norms, (H_1d_) descriptive norms, and (H_1e_) perceived behavioral control, significantly predict the intention to involve oneself in a bullying situation.*

**H_2_.**  *Perceived moral norms significantly predict the intention to involve oneself in a bullying situation, beyond the standard antecedents of the TPB.*

## 2. Materials and Methods

Based on the hypothesized relationships between six independent and one dependent variable, a hierarchical regression analysis [37] and subsequent mediation analyses [38] were conducted in IBM SPSS 28.0.

### 2.1. Participants and Procedure

Data were collected from undergraduate university students in the United Kingdom using a digital questionnaire. The dataset was obtained using the commonly used scientific research participant recruitment platform, Prolific.co.uk [39,40]. All participants were provided with a small monetary compensation for their participation. The questionnaire was designed using SurveyXact, and the data collection took place in October 2022, when most UK universities resumed in-person classes after the main period of the COVID-19 pandemic. The study was approved by the Norwegian National Agency for Research Data, ensuring compliance with methodological, ethical, and data processing guidelines. All participants were informed about the purpose of the study, their right to withdraw their information at any point, and the processing of their data. They provided digital consent to participate. 

A pilot sample consisting of *N* = 49 participants was gender-balanced. Initially, fifty participants were included in the pilot sample; however, one participant experienced a technical error and skipped the final mandatory items regarding the covariates. As a result, this participant was excluded from the pilot sample once the missing data were identified. Participants in both the pilot and the main sample could openly comment on the questionnaire’s comprehensiveness in an optional open-text field. The main study included the pilot sample and consisted of *N* = 419 participants (*M_age_* = 22.76, *SD_age_* = 4.02, 47.0% male, 50.6% female, 2.4% other). Since Prolific.co.uk continuously invites eligible participants into the study until the agreed sample size is reached, making assumptions on the response rate for this study is not possible. One participant did not indicate their age, and participants aged between 18 and 35 years were invited to participate in the study. Among the participants, 86.6% were of British nationality, and 34 other nationalities were represented. Ethnicity was obtained in a simplified manner, with participants indicating themselves to be 16.7% Asian, 4.8% Black, 3.8% mixed ethnicity, 73.3% white, and 1.4% other ethnicities. All participants self-reported fluency in English and were enrolled as undergraduate students at a university in the United Kingdom. 

### 2.2. Vignette

A vignette design was selected, and the participants read the following text:

Imagine the following situation:

As part of a course at your university, you (D) are obliged to conduct a graded group project with three other students. Your work has to be finished in two weeks’ time, and you started the collaboration about a week ago. Group members A and B are close friends, and you and the other group member C have been randomly assigned to the group. While you get along well with A and B, they have been treating C differently from the beginning. When C makes constructive comments or suggestions during the group work, A and B often degrade or question these. When C then asks questions or tries to continue the discussion, A and B ignore C. It has also happened that A and B did not send C some requested materials within an agreed time. After your work is finished, A and B decide to have lunch together, and they invite only you to join them. C notices your conversation while everyone is packing their stuff. 

**Figure 1:**
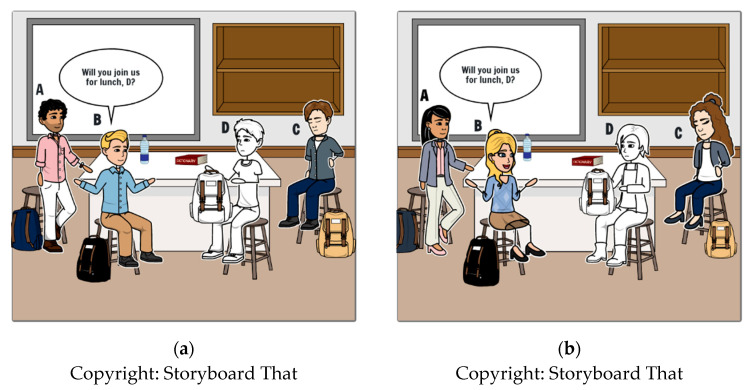
(**a**) Illustration indicating male students; (**b**) illustration indicating female students.

Hence, four students and their roles were described: two students, A and B, who engage in excluding another student, C, and the bystander, D, whose perspective the respondents were asked to consider. A vignette was accompanied by a cartoon illustration. Following the approach employed by Brody and Vangelisti [12] in their vignette-based study, group effects between genders were minimized to a feasible extent by presenting respondents with a cartoon illustration, indicating students of their self-reported gender (Figure 1a,b). Participants who identified as “other” were presented with one of the two versions randomly. The text and illustration were consistently displayed throughout the assessment of every scale that referred to the same hypothetical situation, ensuring that the respondents consistently related to the same scenario. 

### 2.3. Measures

#### 2.3.1. Standard Theory of Planned Behavior Variables

The standard TPB variables, including intention, cognitive attitudes, emotional attitudes, injunctive norms, and descriptive norms, were specifically designed for the context of this study and the vignette employed. The variable measuring perceived behavioral control consisted of items adapted from different studies, as described below. 

The behavioral intention to involve oneself in the situation was measured using four items corresponding to the question “How much do you agree with the following: ‘I would interfere in the situation shown above’, ‘I would involve myself in the situation shown above’, ‘I would help resolve the situation shown above’, ‘I would do something to stop the situation shown above’?” Participants indicated their agreement on a five-point Likert scale ranging from 1 = Strongly disagree to 5 = Strongly agree (M = 4.00, SD = 0.84, Cronbach’s α = 0.90). 

Emotional attitudes and cognitive attitudes toward the behavior in question were each assessed using three items, following the stem “Involving myself in a situation as shown above would be...”. Participants responded on a scale ranging from 1 = Completely worthless to 6 = Completely valuable, 1 = Completely unwise to 6 = Completely wise, and 1 = Completely bad to 6 = Completely good for cognitive attitudes (M = 4.54, SD = 0.93, Cronbach’s α = 0.84) and 1 = Completely wrong to 6 = Completely right, 1 = Completely frightening to 6 = Completely safe, and 1 = Completely demanding to 6 = Completely effortless for emotional attitudes (M = 3.28, SD = 0.89, Cronbach’s α = 0.72).

Subjective norms were composed of injunctive norms (social pressure) and descriptive norms (assumptions about others’ behavior). Each construct was measured using two items each. The items were ‘People who are important to me expect me to do something about a situation as shown above’ and ‘People who are important to me expect me to respond to a situation as shown above’ for injunctive norms and ‘People who are important to me would do something if they had been in a situation as shown above’ and ‘People who are important to me would react to a situation as shown above’ for descriptive norms. The respondents indicated their agreement on a five-point Likert scale ranging from 1 = Strongly disagree to 5 = Strongly agree for both injunctive norms (M = 4.03, SD = 0.73, Spearman’s ρ = 0.74 with *p* < 0.01) and descriptive norms (M = 3.91, SD = 0.78, Spearman’s ρ = 0.60 with *p* < 0.01).

Perceived behavioral control was measured using five items. As most studies present only one or two items to measure perceived behavioral control, an expanded five-item scale was used by combining items from several studies, following the prompt “How much do you agree with the following?” The items ‘Involving myself in a situation as shown above would be difficult’, ‘I feel that I am able to involve myself in a situation as shown above’ [41], ‘It is mostly up to me whether I involve myself in a situation as shown above’, and ‘I have complete control over whether I involve myself in a situation as shown above’ [14] were complemented by a fifth item, ‘Involving myself in a situation as shown above would be easy’. All items were rated on a seven-point Likert scale ranging from 1 = Strongly disagree to 7 = Strongly agree (M = 4.93, SD = 1.03, Cronbach’s α = 0.76).

#### 2.3.2. Perceived Moral Norms as an Additional Variable

To measure the students’ perceived moral norms, seven items from the Moral Norms scale developed by de Leeuw et al. [41] were adapted and adjusted to the bullying context as necessary. The participants responded to four items following the stem: “How much do you agree with the following? If I involved myself in a situation as shown above… ‘I would show respect to C’, ‘I would be a responsible person’, ‘I would feel that I did something morally right’, and ‘I would have a good conscience’”. The remaining three items were ‘I feel morally obligated to involve myself in a situation as shown above’, ‘My personal values lead me to involve myself in a situation as shown above’, and ‘I think involving myself in a situation as shown above is the right thing to do’. The respondents indicated their agreement with all seven items on a six-point Likert scale ranging from 1 = Definitely not to 6 = Yes, definitely (M = 5.13, SD = 0.70, Cronbach’s α = 0.88).

#### 2.3.3. Sociodemographic Variables and Covariates

Gender was assessed using the item “Which gender describes you best?” and 1 = male, 2 = female, and 3 = other were ascribed. In addition, respondents aged between 18 and 35 years were invited to participate in the study, and their ages were retrieved from https://www.prolific.co/.

When individuals are asked about their attitudes, behavior, or personal characteristics, response biases can occur in self-reported data, leading to distorted results that may not accurately reflect reality to the extent feasible in methodological terms. One such response bias is socially desirable responding, which occurs when individuals have a tendency to provide positive descriptions of themselves that may contradict reality [42]. Socially desirable responding consists of a gamma factor that follows a moralistic bias and is expressed through the portrayal of “saint-like” attributes and self-deceptive denial, such as emphasizing traits like dutifulness, restraint, or agreeableness. The present study employed a short scale from Kemper et al. [43] to measure the moralistic bias of socially desirable responding. The scale comprises two subscales for the exaggeration of positive qualities and the understatement of negative qualities. Following the prompt “The following statements may apply more or less to you personally. Please indicate to what extent they apply to you”, the respondents were presented with three items for an exaggeration of positive qualities, ‘In an argument, I always remain objective and stick to the facts’, ‘Even if I am feeling stressed, I am always friendly and polite to others’, and ‘When talking to someone, I always listen carefully to what the other person says’, and three items for an understatement of negative qualities, ‘It has happened that I have taken advantage of someone in the past’, ‘I have occasionally thrown litter in the countryside’, and ‘Sometimes I only help people if I expect to get something in return’. Three items were rated on a five-point scale ranging from 1 = Doesn’t apply at all to 5 = Applies completely for both an exaggeration of positive qualities (M = 3.60, SD = 0.72, Cronbach’s α = 0.64) and understatement of negative qualities (M = 1.81, SD = 0.70, Cronbach’s α = 0.58).

To determine how relevant this study was to students’ lives and their ability to imagine the situation described in the vignette, one item assessed their experience with social exclusion as bystanders: “How often have you experienced that someone was singled out by others in your everyday study?” All 419 participants responded to this item, and the results indicated that 13.6% had never experienced such a situation, 33.9% had experienced it seldom, and 44.4% had sometimes experienced it. In addition, 28 students had experienced this kind of situation at least 1–2 times a week (6.7%), and 6 participants experienced it daily (1.4%). Hence, 86.4% of the students had witnessed social exclusion among their peers in the past, with about half of them experiencing it sometimes or more frequently. These findings suggest that most of the participants were able to realistically imagine a hypothetical situation based on their own experiences. 

### 2.4. Data Analysis

A hierarchical regression analysis [37] was carried out using IBM SPSS 28.0, with the intention to involve oneself in the bullying situation as the dependent variable, with (1) covariates of gender, age, exaggeration of positive qualities, and understatement of negative qualities, (2) the standard TPB constructs of cognitive attitudes, emotional attitudes, injunctive norms, descriptive norms, and perceived behavioral control, and (3) perceived moral norms as additional predictors. No violation of linear relationships was detected, scatterplots indicated the multivariate normality of residuals and homoscedasticity, and no multicollinearity was observed, with a VIF value below 3.00 for all of the independent variables. 

In Model 1, age, gender, and the two scales for SDR were entered as control variables. In Model 2, all standard TPB variables (i.e., cognitive attitudes, emotional attitudes, injunctive norms, descriptive norms, and perceived behavioral control) were entered. Since the results from Model 2 opposed the TPB, subsequent analysis was conducted in smaller steps, separately focusing on the dimensions of attitudes, norms, and mediation effects. Mediation may be assumed when the relationship between an independent and dependent variable in a multivariate regression analysis is reduced by a third (mediating) variable [38]. This assumption is typically made in two scenarios. First, it occurs when the hypothesized effect is not observed immediately upon introducing the independent variable into the model, resulting in non-significant findings. Second, it arises when a previously significant regression coefficient is reduced or becomes non-significant once an additional variable is introduced in a hierarchical regression analysis [38]. After excluding the possibility of mediation effects among the standard TPB antecedents, the initially planned model was calculated. Finally, mediation analyses were conducted via the PROCESS macro for IBM SPSS 28.0 [38] using ordinary-least-squares-regression-based path analysis to explore the relationships between the significant TPB variables in Model 3 (i.e., cognitive attitudes, descriptive norms, and perceived behavioral control), with perceived moral norms as the mediating variable.

## 3. Results

### 3.1. Descriptive Statistics and Pearson Correlations

Descriptive statistics, such as means and standard deviations, reliability coefficients, and Pearson correlation coefficients among all variables are presented in Table 1. Given the mandatory responses, the dataset was complete, with no missing data. Consistent with the above-mentioned theoretical propositions, all independent variables were significantly correlated with the dependent variable. The covariates age and gender and the two variables for socially desirable responding were also significantly correlated with the intention to involve oneself, which was in line with the assumption that a moralistic bias should be considered in the subsequent hierarchical regression analysis. Perceived moral norms, the extension to the standard TPB variables, were significantly correlated with the TPB variables, with significant correlation coefficients of *r* > 0.50 and *p* < 0.01, except for emotional attitudes (*r* = 0.17, *p* < 0.01).

### 3.2. Hierarchical Regression Analysis

In Model 1, age (*β* = 0.11, *p* = 0.02), gender (*β* = 0.12, *p* < 0.01), and exaggeration of positive qualities (*β* = 0.18, *p* < 0.001) significantly predicted the intention (*R*^2^*_Adj_* = 0.08). In Model 2, in line with H_1b_, H_1d_, and H_1e_, cognitive attitudes (*β* = 0.39, *p* < 0.001), descriptive norms (*β* = 0.14, *p* < 0.01), and perceived behavioral control (*β* = 0.30, *p* < 0.001) significantly predicted the intention. However, H_1a_ and H_1c_ were not supported by the data, as neither emotional attitudes nor injunctive norms significantly predicted the dependent variable (*R*^2^*_Adj_* = 0.44). The results from Model 2 indicated that cognitive attitudes may mediate the effect of emotional attitudes on intention, since emotional attitudes did not significantly predict intention (*R*^2^*_Adj_* = 0.44). Therefore, only emotional attitudes and cognitive attitudes were entered into Models 2 and 3, respectively. Moreover, as emotional attitudes were not able to significantly predict the intention without including cognitive attitudes in the regression model, no mediation occurred between the two constructs of emotional attitudes and cognitive attitudes. Hence, both variables were treated as initially planned. Second, the results from Model 2 indicated that the effect of injunctive norms on the intention might be mediated by descriptive norms, since only descriptive norms significantly predicted the intention. Thus, only the injunctive norms and descriptive norms were entered in Models 2 and 3, respectively. Nevertheless, when entered alone in Model 2, injunctive norms (*β* = 0.06, *p* = 0.13) did not significantly predict the intention (*R^2^_Adj_* = 0.43) with the other variables entered. Mediation could not be assumed for the relationship between injunctive norms and descriptive norms and the intention. 

The Initially planned model was calculated, and the results are shown in Table 2. In the final Model 3, perceived moral norms were added as an extension to the standard model of the TPB and, in line with H_2_, significantly predicted the intention (*β* = 0.30, *p* < 0.001, *R*^2^*_Adj_* = 0.47), alongside cognitive attitudes (*β* = 0.29, *p* < 0.001), descriptive norms (*β* = 0.10, *p* < 0.05), and perceived behavioral control (*β* = 0.21, *p* < 0.001). Simultaneously, changes in other significant standardized coefficients were observed. Perceived moral norms not only significantly predicted the intention but also possibly interacted with other predictors of the intention with cognitive attitudes (Δβ = −0.10), descriptive norms (Δβ = −0.04), and perceived behavioral control (Δβ = −0.09).

### 3.3. Mediation Analyses

**Figure 2:**
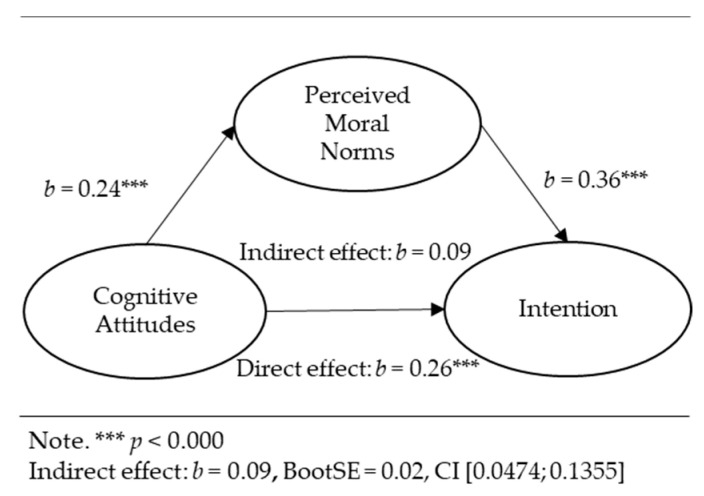
Indirect effect of cognitive attitudes on intention through perceived moral norms.

**Figure 3:**
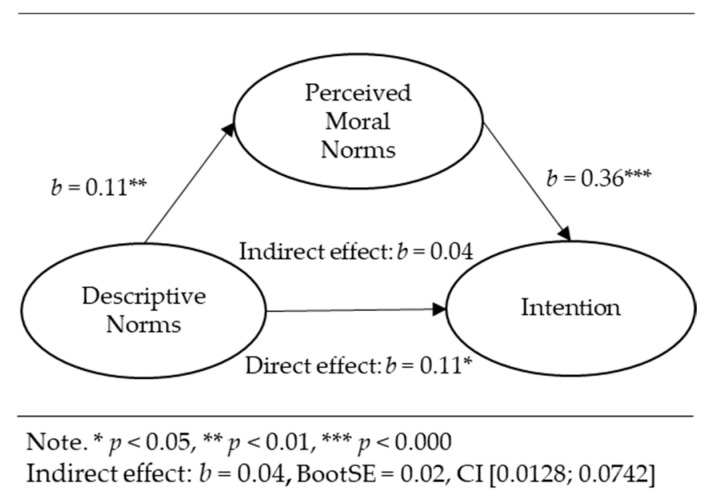
Indirect effect of descriptive norms on intention through perceived moral norms.

**Figure 4:**
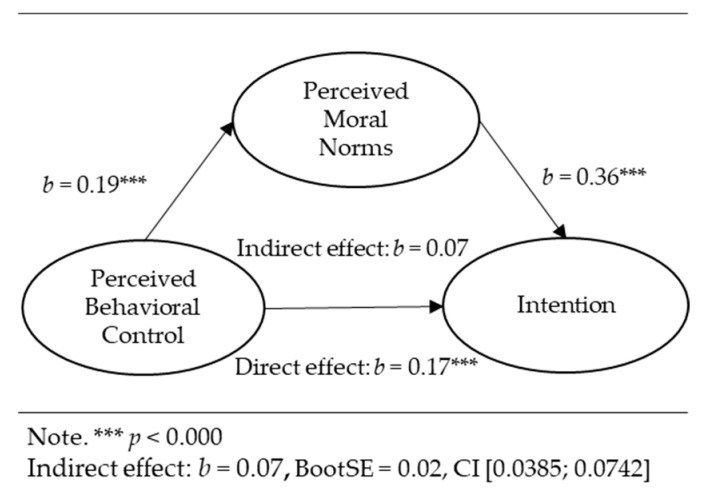
Indirect effect of perceived behavioral control on intention through perceived moral norms.

Mediation analyses for the relationships between perceived moral norms, cognitive attitudes, descriptive norms, perceived behavioral control, and the intention were conducted via the PROCESS macro for IBM SPSS 28.0 [38] using 5000 samples for percentile bootstrap with a 95% confidence interval. The following results are graphically presented in Figure 2, Figure 3 and Figure 4. The results below represent the unstandardized regression coefficients (*b*). The standardized regression coefficients (*β*) between the independent variables and covariates and the intention as a dependent variable were equal to those in the hierarchical regression analysis (Table 2). Perceived moral norms significantly positively predicted the intention while controlling for cognitive attitudes, descriptive norms, perceived behavioral control, and other predictors (*b* = 0.36, *p* = 0.000).

Cognitive attitudes significantly predicted perceived moral norms (*b* = 0.24, *p* = 0.000). The bootstrap confidence interval for the indirect effect of cognitive attitudes (*b* = 0.09, *Boot SE* = 0.02) was 0.0474–0.1355, indicating the indirect effect of cognitive attitudes on the intention through perceived moral norms. Cognitive attitudes had a direct effect on the intention (*b* = 0.26, *p* = 0.000).

Descriptive norms were positively related to perceived moral norms (*b* = 0.11, *p* < 0.01). The bootstrap confidence interval for the indirect effect of descriptive norms (*b* = 0.04, *Boot SE* = 0.02) was 0.0128–0.0742, suggesting the indirect effect of descriptive norms on the intention through perceived moral norms. Descriptive norms had a direct effect on the intention (*b* = 0.11, *p* < 0.05).

Perceived behavioral control was positively related to perceived moral norms (*b* = 0.19, *p* = 0.000). The bootstrap confidence interval for the indirect effect of perceived behavioral control (*b* = 0.07, *Boot SE* = 0.02) was 0.0385–0.1032, showing the indirect effect of perceived behavioral control on the intention through perceived moral norms. Perceived behavioral control had a direct effect on the intention (*b* = 0.17, *p* = 0.000).

Partial mediation for the effects of each standard TPB antecedent (i.e., cognitive attitudes, descriptive norms, and perceived behavioral control) significantly predicting the intention through the mediator variable of perceived moral norms is evident.

## 4. Discussion

The present study examined the utility of the TPB and perceived moral norms as an additional variable in predicting university students’ intentions to involve themselves in a situation of social exclusion as bystanders. The intention was significantly predicted according to cognitive attitudes, descriptive norms, perceived behavioral control, and perceived moral norms. Contrary to the assumptions of the TPB, emotional attitudes and injunctive norms did not significantly predict the intention. Furthermore, perceived moral norms mediated the relationships between cognitive attitudes, descriptive norms, and perceived behavioral control as the independent variables and the intention as the dependent variable. This mediation effect was small yet statistically significant.

### 4.1. Moralistic Bias in Quantitative Research on Bystander Intentions

Age, gender, and the exaggeration of positive qualities as a subscale of socially desirable responding were significant predictors of the intention when entered as covariates. While age and gender are common sociodemographic variables to control for, socially desirable responding is seldom included in analyses of bystander intentions, even though the positive presentation of oneself could play a considerable role in situations leading to a moral dilemma, such as relational bullying. However, the exaggeration of positive qualities as a subscale for socially desirable responding could initially account for a proportion of the variance in intention as a significant predictor. Moreover, the TPB standard variables could explain a much greater proportion of the explained variance when entered, and the exaggeration of positive qualities was no longer a significant predictor of the intention. This finding confirms that the participants would not report a prosocial intention to involve themselves in social exclusion based on a moralistic bias solely because society would wish for them to do so, but that reporting the intention does in fact result from their attitudes, norms, and perceived behavioral control.

### 4.2. Standard Theory of Planned Behavior Antecedents Related to Bystander Intentions

Contrary to the assumptions of the TPB and H_1a_, emotional attitudes did not significantly predict the intention. According to other theories on behavioral reasoning, actions to support another individual in need are shaped by both cognitive and affective processes, such as the assessment of costs and rewards and arousals or emotions [44]. For example, individuals who are part of a group, and who hold strong attitudes toward the correct types of behavior within this group, may attempt to lead the group in moments of nonconformity to group norms shown by other members [45]. Hence, cognitive attitudes and emotional attitudes are assumed to affect the bystander’s intentions toward a group situation in which other members do not comply with the bystander’s attitudes and perceived group norms. In this case, cognitive attitudes were the only significant predictor of the intention, supporting H_1b_. Perhaps the represented scenario was not able to fully tap into an individual’s emotions due to its low stressor impact based on a certain emotional distance created by a hypothetical scenario presented online, making it a more cognitive–affective process [44]. However, this would be rather surprising, since social exclusion observed in somebody else relates to feelings of belongingness and social value and should therefore appeal to one’s emotional attitudes toward social exclusion and intentions toward it [46]. Consequently, the items developed toward emotional attitudes may not fully reflect the intended construct due to unsuitable items.

Unlike descriptive norms, injunctive norms were not able to significantly predict the intention, not supporting H_1c_. Following other prominent theories on behavioral reasoning, actions and articulations by individuals communicate group norms, thereby relating to interpersonal actions [44,47,48,49]. Solidarity, as one component of identification with other group members, may further create a psychological bond with the group, thus determining an individual’s group-based activities [50]. Hence, injunctive norms and descriptive norms perceived by bystanders in relation to their peer group may be associated with how individuals intend to behave both toward and in social exclusion situations. A reason for the missing association between injunctive norms and intention lies in the complexity of the measure, as injunctive norms are measured as the respondent’s assumptions about others’ expectations toward their own hypothetical behavior. In contrast, descriptive norms are straightforward assumptions about other people’s hypothetical behavior and significantly predict the intention, supporting H_1d_. Therefore, they are easier to estimate based on experience and follow-up. Hence, the operationalization of injunctive norms calls for further investigation in future research into the TPB and social exclusion. In support of H_1e_, perceived behavioral control was the third standard TPB variable that significantly predicted the intention to involve oneself in the bullying situation.

TPB studies commonly only partially support the TPB’s assumptions. For instance, in studies on assertive bystanders and bullying intentions among college students, perceived behavioral control was not a significant predictor of intention [14,51]. In another study, neither injunctive nor descriptive norms significantly predicted intention; however, a combined construct, including moral norms, did [52]. Although the TPB extended by moral norms is widely accepted as an applicable framework for predicting various behavioral intentions, varying circumstances may still lead to deviations from the assumed outcomes [29].

### 4.3. Moral Norms as an Additional Predictor of Bystander Intentions

In line with H_2_, perceived moral norms were a significant predictor of the intention when added to the standard antecedents of the TPB. This supports the assumption that perceived moral norms can significantly contribute to the prediction of behavioral intention when the welfare of others is at stake [28]. The results also indicated low mediation effects between perceived moral norms and the three significant predictors of the intention (i.e., cognitive attitudes, descriptive norms, and perceived behavioral control), as the effects of the standard antecedents on moral norms were similar to the direct effects of the standard antecedents on the intention. A plausible reason for the mediation of the relationship between cognitive attitudes and the intention through perceived moral norms could lie in the conceptual similarities between both constructs, indicating that perceived moral norms could represent features of cognitive attitudes, and vice versa, leading to a possible conceptual overlap [53]. The indirect effect of descriptive norms on the intention through perceived moral norms could, as the names suggest, be based on the normative characteristics of the constructs constituted by the social group and mutual behavioral expectations. Observing others acting in moral dilemmas influences an individual’s normative evaluation of handling such situations when deciding which action would be right or wrong to take. Considering the indirect effect of perceived behavioral control on intentions through perceived moral norms, one’s conviction that an action is feasible may influence whether acting is considerable from a moral point of view. Once an individual has decided that an action is feasible and all resources, time, skills, and knowledge are considered, moral normative evaluation occurs and allows for the decision as to whether other factors in one’s value system hinder the action. After both evaluations on perceived control and moral aspects have taken place, the individual may then possibly form an intention to involve oneself in a moral dilemma, such as a situation of bullying among peers. Hence, social exclusion as a form of bullying is indicative of an individual’s perceived control and moral aspects.

### 4.4. Limitations and Further Research

One of the limitations of this study is the widespread problem of using self-reported data, which always underlies the respondents’ subjective interpretation of their own and others’ beliefs and behaviors. Moreover, the data on respondents’ intentions toward a hypothetical situation only provide an indication of university students’ actual behavior toward social exclusion. The present study assessed only the intention to involve oneself assertively, whereas bystanders have a range of behaviors to choose from when reacting to social exclusion [15]. Future research should assess various behaviors as dependent variables and include both prosocial and antisocial intentions [33,54,55,56]. As the situation presented in the vignette represents only one of the numerous potential scenarios that could happen among university students, changes in the vignette could lead to changes in the outcome of the study, requiring more similar research to cover the situational dimension of bystanders’ intentions.

Furthermore, the study was conducted via the facilitating platform Prolific.co.uk, which not only led to a potentially biased sample who might have had a special interest in the topic of bullying or social exclusion but also meant that a specific group of respondents were represented (university students in the United Kingdom, predominantly of British nationality), thus calling for further research targeting other populations to compare results and allow for conclusions on their transferability. The participants’ age, gender, English proficiency, and student status were, as with all other variables, self-reported. The correctness of this information is therefore trust-based; however, https://www.prolific.co/ allows people of all professional and educational statuses to register on the platform and participate in surveys directed at various target groups. Thus, the participants in this study may in fact have been students. Regarding the sample size, the present study was not conducted with the aim of generalizability. Findings from this first multi-university sample of 419 participants can nevertheless provide important guidance for future research.

Regarding potential group effects, group identification, friendships, and different appraisals of the need to belong can be considered in future research [12,45,46]. The present study was designed with gender-based illustrations to reduce the influence of gender; however, highly complex factorial designs allow for a thorough investigation of the effect of gender on bystanders’ intentions, depending on the respondents’ and fictive characters’ genders. Other considerable factors include sexual orientation, socioeconomic status, ethnicity, and relationships within the classroom [12,57,58]. Moreover, expanded sample sizes and multiple vignettes can allow for the examination of in-group and out-group effects of, for example, ethnicity on the study outcome.

### 4.5. Implications

Notwithstanding these limitations, the present study offers some valuable empirical, theoretical, and practical implications for professionals, aiming to design prevention and intervention programs aimed at HE institutions to foster prosocial bystander reactions toward social exclusion. On an empirical level, the results indicate that not all of the included constructs were operationalized to their best potential, calling for a further investigation of designing a TPB questionnaire for bystander scenarios in which attitudes and norms are divided into sub-constructs, emotional and cognitive attitudes, and injunctive and descriptive norms. Many of the TPB studies reviewed for the present paper did not distinguish between emotional and cognitive attitudes, although the TPB implies this. This factor might be due to various reasons and simply lie in the length of the questionnaire or the context under investigation; however, the difficulty of operationalization might be another reason for not including emotional attitudes as a construct. Therefore, the distinction between emotional and cognitive attitudes should be investigated further on an empirical level to determine whether the issue is of an empirical or theoretical nature.

The successful inclusion of moral norms as an additional variable reflects the results of various previous studies and supports Ajzen’s thesis, stating that moral norms should be included in TPB studies that have a clear moral dimension to them [30]. Previous research has found that moral norms are determined by laws, parents, peers, and religion, suggesting that HE institutions ought to promote collective responsibility toward prosocial bystander intervention [52]. Raising awareness of bystanders’ responsibility and their behaviors’ moral impact can be crucial for social developments in educational environments [59]. Research on moral development underlines the importance of moral education from a lifelong perspective, beyond secondary education into HE and work-life [60,61,62]. Furthermore, the related construct of moral courage has been associated with bystanders’ self-competence and self-efficacy [17], whereby the latter is conceptually closely linked to perceived behavioral control [63].

The findings of the present study have some valuable implications for the design of intervention campaigns against bullying and for bystander involvement. A meta-analysis on the effectiveness of intervention programs on various bullying types confirmed that overall, interventions against relational bullying are effective [64]. As shown in another meta-analysis [65], the TPB offers a reliable theoretical framework to design interventions addressing the encouragement of intentions. Within the topic of bystanders, some studies have supported intervention programs that directly apply the TPB [13,66,67], whereas a meta-analytic review has shown that interventions that apply only individual antecedents of the TPB have been successfully employed in educational settings [68]. For instance, an American university successfully rolled out the It’s Your Place campaign to encourage prosocial bystander intervention by students who witnessed indications of upcoming or already-happening sexual assault among other students [67].

For practitioners in HE, the present study indicates that fostering prosocial bystander behaviors in university students ought to be based on students’ attitudes toward involving themselves in a bullying situation, students’ mutual expectations and normative views on their involvement, and the development of constructive student intervention strategies to encourage perceived control over their own behavior in these situations. For example, practitioners can bring together students to discuss bullying scenarios and support their problem-solving skills [69]. By addressing moral dilemmas, students can reflect on how to negotiate and overcome them [70]. This could prevent the negative consequences of immoral standpoints on bystander involvement, as some students reported, for example, that they see that the victim is an adult and should be able to help themselves, that they do not want to become involved in other people’s business, or that they do not want to risk worsening the situation for the victim in case they, as bystanders, intervened in the social exclusion situation [17]. Furthermore, lecturers and professors at universities should consider how much space of engagement individuals and groups within their classrooms occupy to facilitate and ensure a balanced classroom climate in which all students feel heard and acknowledged [71].

In many countries, including the United Kingdom, universities are legally obliged to protect students from bullying and other harmful incidents or treatments [15]. Previous studies have indicated that students did not know whom to report bullying to at their institution, suggesting low reporting rates of bullying in HE [7,69]. One possible reason for this may be that students do not have sufficient knowledge on how to identify bullying, which in turn calls for clear communication of bullying definitions, examples, and guidance for reporting and support services [72]. To prevent bullying, widespread efforts to challenge norms of minimization and justification of bullying are recommended [15]. To raise awareness of bullying in HE and foster prosocial bystander encouragement in universities, policy makers should define the accountabilities of institutions, researchers, and funding agencies as the main stakeholders [73]. Proposed measures for tackling bullying in academia include the creation of centers of excellence researching (in)civility and ethics in the academic environment and the inclusion of bullying prevalence in university rankings [73].

## 5. Conclusions

In conclusion, the TPB provides an adequate theoretical framework for the prediction of university students’ prosocial bystander intentions toward social exclusion as a form of relational bullying. Although not all independent variables significantly predicted the intention, cognitive attitudes, descriptive norms, and perceived behavioral control were, nevertheless, able to account for a significant proportion of the explained variance in intention. Moreover, this study showed that moral norms may significantly enhance the proportion of explained variance in the intention to involve oneself in a situation of social exclusion and contribute to its prediction beyond the standard TPB antecedents. Thus, the present study has provided valuable knowledge to educators in HE, university psychologists or mental health counselors, policy makers, and greater society. The implications can be especially useful for professionals aiming at designing prevention and intervention programs in HE institutions to foster prosocial bystander reactions toward social exclusion as a form of bullying.

## Figures and Tables

**Table 1:** Descriptive statistics and Pearson correlation matrix.

	1	2	3	4	5	6	7	8	9	10	11
1. INT	1										
2. Gender	0.16 **	1									
3. Age	0.14 **	0.09	1								
4. SDR (PQ+)	0.21 **	0.03	0.06	1							
5. SDR (NQ−)	−0.16 **	−0.25 **	−0.04	−0.15 **	1						
6. CA	0.55 **	0.16 *	−0.01	0.14 *	−0.13 *	1					
7. EA	0.23 **	0.00	0.05	0.09	−0.01	0.24 **	1				
8. IN	0.35 **	0.12 *	0.02	0.17 **	−0.07	0.40 **	0.08	1			
9. DN	0.42 **	0.08	0.10 *	0.15 **	−0.10 *	0.38 **	0.18 **	0.51 **	1		
10. PBC	0.49 **	0.07	0.13 *	0.22 **	−0.10 *	0.33 **	0.49 **	0.35 **	0.39 **	1	
11. PMN	0.61 **	0.20 **	0.13 *	0.27 **	−0.27 **	0.57 **	0.17 **	0.56 **	0.50 **	0.52 **	1
*M* (*SD*)	4.00 (0.84)	-	22.76 (4.02)	3.60 (0.72)	1.81 (0.70)	4.54 (0.93)	3.28 (0.89)	4.03 (0.73)	3.91 (0.78)	4.93 (1.03)	5.13 (0.70)
Reliability	*α* = 0.90	-	-	*α* = 0.64	*α* = 0.58	*α* = 0.84	*α* = 0.72	*ρ* = 0.74 **	*ρ* = 0.60 **	*α* = 0.76	*α* = 0.88

Notes. *N* = 419. INT = intention, SDR = socially desirable responding, PQ+ = exaggeration of positive qualities, NQ− = understatement of negative qualities, CA = cognitive attitudes, EA = emotional attitudes, IN = injunctive norms, DN = descriptive norms, PBC = perceived behavioral control, MN = perceived moral norms. * Correlation is significant at the 0.05 level (two-tailed). ** Correlation is significant at the 0.01 level (two-tailed).

**Table 2:** Hierarchical linear regression predicting intention.

Variable	*B*	*SE B*	*β*	*t*	*R* ^2^ *_Adj_*	*F_Change_*
Model 1					0.08	9.92 ***
Gender	0.19	0.08	0.12 *	2.51		
Age	0.02	0.01	0.11 *	2.38		
SDR (PQ+)	0.21	0.06	0.18 ***	3.85		
SDR (NQ−)	−0.11	0.06	−0.09	−1.89		
Model 2					0.44	53.68 ***
Gender	0.07	0.06	0.05	1.21		
Age	0.02	0.01	0.09 *	2.27		
SDR (PQ+)	0.07	0.04	0.06	−0.99		
SDR (NQ−)	−0.05	0.05	−0.04	−1.06		
CA	0.35	0.04	0.39 ***	9.10		
EA	−0.04	0.04	−0.04	−0.97		
IN	0.02	0.05	0.01	0.28		
DN	0.15	0.05	0.14 **	3.10		
PBC	0.24	0.04	0.30 ***	6.33		
Model 3					0.48	29.93 ***
Gender	0.05	0.06	0.03	0.89		
Age	0.01	0.01	0.06	1.76		
SDR (PQ+)	0.04	0.04	0.03	0.87		
SDR (NQ−)	0.01	0.05	0.01	0.13		
CA	0.26	0.04	0.29 ***	6.47		
EA	−0.01	0.04	−0.01	−0.27		
IN	−0.07	0.05	−0.07	−1.41		
DN	0.11	0.05	0.10 *	2.30		
PBC	0.17	0.04	0.21 ***	4.44		
PMN	0.36	0.07	0.30 ***	5.47		

Notes. *N* = 419. SDR = socially desirable responding. PQ+ = exaggeration of positive qualities. NQ− = understatement of negative qualities. CA = cognitive attitudes, EA = emotional attitudes, IN = injunctive norms, DN = descriptive norms, PBC = perceived behavioral control, PMN = perceived moral norms. * *p* < 0.05. ** *p* < 0.01. *** *p* < 0.001.

## Data Availability

The raw, anonymized dataset may be requested directly from the author and will be made available after completion of the Ph.D. project, which this study is a part of.
